# *Plasmodium vivax* Relapse Rates Following *Plasmodium falciparum* Malaria Reflect Previous Transmission Intensity

**DOI:** 10.1093/infdis/jiz052

**Published:** 2019-01-29

**Authors:** Elizabeth A Ashley, Aung Pyae Phyo, Verena I Carrara, Kyaw Myo Tun, Francois Nosten, Frank Smithuis, Nicholas J White

**Affiliations:** 1 Myanmar Oxford Clinical Research Unit, Myanmar; 2 Department of Preventive and Social Medicine, Defence Services Medical Academy; 3 Medical Action Myanmar, Yangon, Myanmar; 4 Centre for Tropical Medicine and Global Health, Nuffield Department of Medicine, University of Oxford, United Kingdom; 5 Shoklo Malaria Research Unit, Mahidol-Oxford Tropical Medicine Research Unit, Faculty of Tropical Medicine, Mahidol University, Mae Sot; 6 Mahidol-Oxford Tropical Medicine Research Unit, Faculty of Tropical Medicine, Mahidol University, Bangkok, Thailand

**Keywords:** Malaria, *P. vivax*, *P. falciparum*, relapse

## Abstract

From 2003 through 2009, 687 of 2885 patients (23.8%) treated for *Plasmodium falciparum* malaria in clinical studies in Myanmar or on the Thailand-Myanmar border had recurrent *Plasmodium vivax* malaria within 63 days, compared with 18 of 429 patients (4.2%) from 2010 onward (risk ratio [RR], 0.176; 95% confidence interval, .112–.278; *P* < .0001). Corresponding data from 42 days of follow-up revealed that 820 of 3883 patients (21.1%) had recurrent *P. vivax* malaria before 2010, compared with 22 of 886 (2.5%) from 2010 onward (RR, 0.117; 95% CI, .077–.177; *P* < .0001). This 6-fold reduction suggests a recent decline in *P. vivax* transmission intensity and, thus, a substantial reduction in the proportion of individuals harboring hypnozoites.

## Teaser

Before 2010, 687 of 2885 patients treated for *Plasmodium falciparum* malaria in Myanmar had recurrent *Plasmodium vivax* malaria within 63 days, compared with 18 of 429 from 2010 onward. This 6-fold reduction suggests a recent substantial decline in *P. vivax* transmission and consequent hypnozoite carriage.

In East Asia, *Plasmodium vivax* malaria commonly follows *Plasmodium falciparum* malaria [1–3]. The incidence is much higher than predicted from the prevalence of mixed infections observed by microscopy of blood films or in caught wild anopheline mosquito vectors [4]. Furthermore, the recurrent infections occur at an interval from the initial infection that is exactly the same as the interval between the initial and first relapse of *P. vivax* infection [5]. This consistent observation has led to the hypothesis that people living in a *P. vivax*–endemic area harbor a “bank” of hypnozoites from previous inoculations that can be activated by a stimulus such as acute *P. falciparum* malaria [5]. Alternative theories of the mechanism of *P. vivax* infection relapse after a short latency period include random activation and a clock mechanism similar to that invoked to explain a long latency period [6]. This high rate of sequential infection was of major therapeutic and public health relevance because, with current highly effective artemisinin combination treatments (ACTs), recurrent *P. vivax* malaria was up to 10 times more frequent than recrudescent *P. falciparum* malaria following acute *P. falciparum* malaria. From a public health standpoint, *P. vivax* malaria was therefore by far the main complication of *P. falciparum* malaria. A *P. vivax* recurrence rate of approximately 30% following *P. falciparum* malaria is strong evidence to support giving radical-cure regimens to patients with *P. falciparum*, as well as to those with *P. vivax* malaria [1–3, 7, 8].

If the activation hypothesis [5] is correct, then the proportion of *P. vivax* infections that follow episodes of acute *P. falciparum* malaria should be a function of the preceding (rather than concurrent) *P. vivax* transmission intensity. This is supported by the relative rarity of the occurrence of *P. vivax* malaria following *P. falciparum* malaria reported among returned travelers from Asia who presumably received one or few infected inocula. It is also supported by the very high proportion of relapses involving genetically related parasites among infants following their first *P. vivax* infection, compared with the much lower proportion among their mothers, in whom, presumably, previously acquired hypnozoites had been activated [9]. It follows that if malaria transmission is reduced, the sequential infection rate should fall, too. We tested this hypothesis in studies conducted in Myanmar, mainly in the east of the country, where village health workers have received support to diagnose and treat malaria and, in some parts, mass treatments have been conducted in malaria hot spots. As a consequence, there has been a recent substantial reduction in the incidence of *P. falciparum* malaria. The reduction in malaria incidence was earliest at the Thailand-Myanmar border [10].

## METHODS

We searched the WorldWide Antimalarial Resistance Network Clinical Trials Publication library, a compilation of antimalarial clinical efficacy trials published between 1960 and 2016, for eligible studies [9]. A search of more-recent studies was made in PubMed, using the search terms “malaria AND Myanmar” with date limits of January 2016 to 2 October 2018. Studies of antimalarial drug efficacy in patients with either uncomplicated *P. falciparum* malaria or mixed malaria parasite infections published since 2005 were eligible if the treatments were standard or extended doses of artesunate or an ACT and patients were followed for at least 28 days. The ACTs were artesunate-amodiaquine, artesunate-mefloquine, dihydroartemisinin-piperaquine, artesunate-pyronaridine, or artemether-lumefantrine (currently recommended first-line treatment in Myanmar). For multicenter studies that did not report *P. vivax* relapse numbers by site, individual-patient data were requested for the study component in Myanmar or on the Thailand-Myanmar border. Unpublished surveillance and study data collected at the Thailand-Myanmar border by the Shoklo Malaria Research Unit over this period were also assessed.

The following variables were extracted into a spreadsheet (Microsoft Excel) by 2 reviewers (E. A. A. and A. P. P.), independently: first author name; PubMed identifier; year of publication; year(s) of study; location; treatment arms; number of *P. falciparum* malaria cases per arm; number of mixed-parasite infections, if reported; and number of *P. vivax* episodes during 28, 42, and 63 days of follow-up. *P. vivax* malaria rates during follow-up were calculated as the number of episodes divided by the number of patients recruited, because reporting of censored patients before the end of follow-up was variable.

## RESULTS

We screened 16 published trial reports. One was excluded because there was no information about *P. vivax* malaria. Patients were enrolled between 2003 and 2016. Data from routine surveillance (during 2010–2011) and 1 unpublished study (prematurely terminated because of low patient numbers after 76 patients had been recruited between 2013 and 2014) collected at the Thailand-Myanmar border by the Shoklo Malaria Research Unit were also included because collection of these data was performed using the same standard methods as in the published clinical trials. The duration of follow-up varied between 28 and 63 days. Studies took place in 7 different states or regions in Myanmar. More than half were at the Thailand-Myanmar border (Table 1). The median number of patients per treatment arm was 161 (range, 37–258 patients). The majority of patients (n = 2678) were treated with artesunate-mefloquine, with the remainder treated with DHA-piperaquine (n = 1393) or artemether-lumefantrine (n = 396). In total, 4829 patients were studied.

**Table 1.** *Plasmodium vivax* Infections Following Treatment of Acute *Plasmodium falciparum* Malaria in Therapeutic Studies in Conducted in Myanmar or Along the Thailand-Myanmar Border

Reference^a^	Year(s) of Study	Location(s)	Antimalarial Treatment	Follow-up Duration, d	*P. falciparum* Cases (Mixed)	*P. vivax* Cases During Follow-up, No. (%)	Children Aged <15 y, No. (No. With *P. vivax* During Follow-up)
[11]	2003–2004	Thailand-Myanmar border (Karen/Kayin State)	ASMQ	63	166 (16)	30 (18.1)	63 (NS)
			DP (4 doses)	63	164 (15)	36 (22.0)	51 (NS)
			DP (3 doses)	63	170 (16)	38 (22.4)	47 (NS)
[12]	2003–2004	Rakhine State	DP	42	156 (21)	22 (14.1)	131 (22)
			DP UnS	42	171 (19)	18 (10.5)	139 (18)
			ASMQ	42	162 (23)	25 (15.4)	133 (25)
			ASMQ UnS	42	163 (14)	32 (19.6)	136 (32)
[13]	2004–2005	Thailand-Myanmar border (Karen/Kayin State)	ASMQ FDC	63	251 (22)	85 (34)	77 (NS)
			ASMQ	63	249 (24)	50 (20)	69 (NS)
[14]^b^	2006	Thailand-Myanmar border (Karen/Kayin State)	ASMQ	63	138 (9)	51 (37)	Adults only
			DP	63	258 (20)	77 (30)	Adults only
[15]^b^	2007–2008	Thailand-Myanmar border (Karen/Kayin State)	ASMQ	42	182 (0)	9 (5.0)	Adults only
			AS PYR	42	369 (0)	73 (19.8)	Adults only
[3]	2008–2009	Rakhine, Kachin, and Shan states	ASAQ	63	155 (26)	59 (38.1)	71 (NS)
			AL	63	162 (25)	85 (52.5)	77 (NS)
			ASMQ FDC	63	169 (21)	55 (32.5)	81 (NS)
			ASMQ	63	161 (31)	75 (46.6)	75 (NS)
			DP	63	161 (26)	56 (34.8)	79 (NS)
[16]	2008–2009	Thailand-Myanmar border (Karen/Kayin State)	ASMQ + PMQ^c^	63	150 (NS)	5 (3.3)	Adults only
SMRU DP follow-up (unpublished data)	2010–2011	Thailand-Myanmar border (Karen/Kayin State)	DP	63	53 (1)	5 (9.4)	7 (1)
[17]^d^	2005–2006	Thailand-Myanmar border (Karen/Kayin State)	ASMQ	63	119 (5)	28 (23.5)	25 (7)
	2007–2008	Thailand-Myanmar border (Karen/Kayin State)	ASMQ	63	212 (9)	38 (17.9)	25 (10)
	2009–2010^e^	Thailand-Myanmar border (Karen/Kayin State)	ASMQ	63	200 (12)	36 (18.0)	25 (10)
	2011–2012	Thailand-Myanmar border (Karen/Kayin State)	ASMQ	63	222 (10)	10 (4.5)	47 (2)
	2013	Thailand-Myanmar border (Karen/Kayin State)	ASMQ + PMQ^c^	63	78 (3)	1 (1.3)	23 (1)
[18]	NS	Tanintharyi Region	AS monotherapy	28	52 (0)	25 (47.2)	Adults only
[19]	2011–2013	Bago Region	AS (3 d) + AL (3 d)	42	80 (3)	0 (0)	Adults only
[20]	2012–2013	China-Myanmar border	DP	42	109 (0)	0 (0)	35 (NS)
[21]	2013–2014	Kachin State and Mandalay Region	DP	42	114 (13)	0 (0)	NS
[22]	2013–2015	Karen/Kayin State	AL (3 d) PMQ^c^	42	78 (1)	1 (1.3)	NS
			AL (5 d) PMQ^c^	42	76 (2)	3 (3.9)	NS
SMRU (unpublished data)^f^	2013–2016	Thailand-Myanmar border (Karen/Kayin State)	DP	63	42 (1)	3 (7.1)	13 (0)
		Thailand-Myanmar border (Karen/Kayin State)	ASMQ	63	43 (0)	2 (4.7)	9 (0)

Abbreviations: AL, artemether-lumefantrine; ASAQ, artesunate-amodiaquine; ASMQ, artesunate-mefloquine; DP, dihydroartemisinin-piperaquine; FDC, fixed-dose combination; NS, not stated; PMQ; primaquine; PYR, pyronaridine; SMRU, Shoklo Malaria Research Unit; UnS unsupervised treatment.

^a^Citation information is specified in the Supplementary Materials, unless otherwise indicated.

^b^Data are from patients recruited by the SMRU only.

^c^PMQ was given as a gametocytocidal single dose (0.25 mg/kg).

^d^A 42-d end point was specified in the article, but data from 63 d were available.

^e^Analyzed as pre-2010 data.

^f^Clinical trials registration NCT01640587.

**Figure 1.**
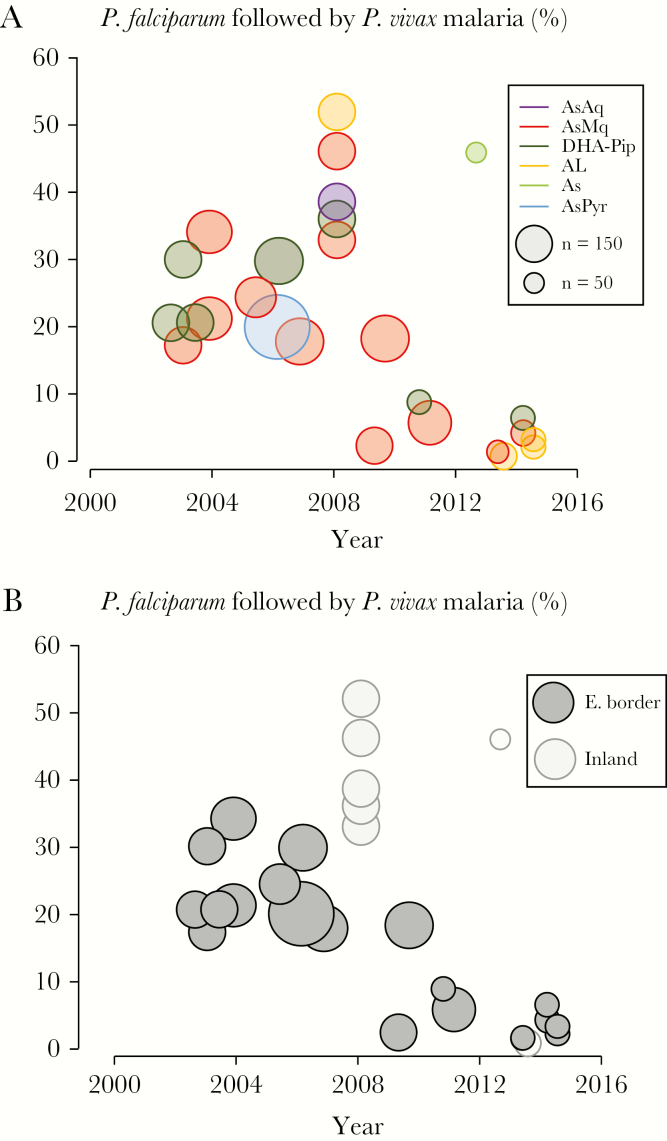
*A*, Proportions of acute *Plasmodium falciparum* malaria episodes that were followed by *Plasmodium vivax* malaria within 9 weeks in each study, by treatment. Studies of artemether-lumefantrine (AL) and artesunate-pyronaridine (AsPyr) with 6 weeks of follow-up and of artesunate monotherapy (As) with 4 weeks follow-up are also included. Studies of dihydroartemisinin-piperaquine (DP) and artesunate-mefloquine (AsMq) with <9 weeks of follow-up were excluded because shorter durations of follow-up (typically ≤ 6 weeks) with these slowly eliminated drugs miss a substantial proportion of relapses. *B*, Proportions of acute *P. falciparum* malaria episodes that were followed by *P. vivax* malaria within 9 weeks in each study, by location (ie, Myanmar-Thailand border vs elsewhere in Myanmar). AsAq, artesunate-amodiaquine.

Rates of *P. vivax* malaria following treatment of uncomplicated *P. falciparum* malaria with artesunate or ACT ranged from 0% to 52.5%. There was a marked recent reduction in the proportion of acute *P. falciparum* malaria cases that were soon followed by *P. vivax* infection (Figure 1A). Before 2010, there were 3883 patients with acute *P. falciparum* malaria in studies with at least 42 days follow-up, of whom 820 (21.1%) had recurrent malaria caused by *P. vivax* within this 6-week period. After 2010, of 886 patients followed for 42 days, only 22 (2.5%) had a *P. vivax* recurrence (risk ratio [RR], 0.117; 95% confidence interval [CI], .077–.177; *P* < .0001). Before 2010, 2885 patients with acute *P. falciparum* malaria had 63 days of follow-up, of whom 687 (23.8%) had recurrent malaria caused by *P. vivax* within this 9-week period. After 2010, of 429 patients followed for 63 days, only 18 (4.1%) had a *P. vivax* recurrence (RR, 0.176; 95% CI, .112–.278; *P* < .0001). In more-recent studies, fewer children were recruited. Otherwise, there were no significant differences in demographic and clinical features between patients with malaria before 2010 and those with malaria during or after 2010.

Most of the studies were conducted along the eastern border with Thailand (in Kayin State), where the SMRU widely deployed ACT via village malaria workers, in addition to insecticide-treated bed nets. From 2007 onward in this location, there was a progressive decline in *P. vivax* malaria recurrences following *P. falciparum* malaria (Figure 1B).

## DISCUSSION

Following the change in Myanmar’s national antimalarial treatment policy to ACT in 2002 and the support for large-scale deployment of ACTs that followed several years later, the incidence of *P. falciparum* malaria declined substantially. Recrudescence rates decreased from >80% in the chloroquine era [12] to <5% with ACTs, but this has revealed a very high rate of *P. vivax* recurrences (presumed to be relapses) in the weeks following treatment [3]. Until recently, approximately one third of patients treated for *P. falciparum* malaria in Myanmar would have a recurrence of malaria within 9 weeks of treatment. In some studies, this proportion reached 50%. Similar results were reported elsewhere in the region [1, 2]. Interestingly, despite the World Health Organization’s current recommendation to change treatment policy for *P. falciparum* malaria when recrudescence rates exceed 10%, no recommendation for radical cure with primaquine was made despite *P. vivax* relapse rates of >30% after *P. falciparum* malaria [1–3, 7]. The interval between acute *P. falciparum* malaria and subsequent *P. vivax* malaria was closely related to the elimination kinetics of the ACT partner drug and was the same as the interval between treatment of primary *P. vivax* malaria and relapse [1–3, 5]. Following artemether-lumefantrine treatment (which has been first-line treatment in Myanmar since 2002 and still provides high cure rates), the sequential *P. vivax* infections usually occurred within 6 weeks after the acute *P. falciparum* malaria episode, whereas the majority of sequential *P. vivax* infections after artesunate-mefloquine and dihydroartemisinin-piperaquine therapy occurred 6–9 weeks afterward [1–3, 5, 7]. *P. vivax* relapse following *P. falciparum* malaria was therefore the principal complication of *P. falciparum* malaria. It was a major cause of morbidity and an important contributor to the overall incidence of *P. vivax* malaria, particularly in adults, and to its transmission.

In this review of almost 5000 prospectively studied patients, approximately one quarter of patients who presented with acute *P. falciparum* malaria before 2010 and were treated with a highly effective ACT had subsequent *P. vivax* malaria within 9 weeks. In studies conducted in early 2009, over half of 162 patients treated with artemether-lumefantrine for *P. falciparum* malaria had *P. vivax* recurrences, yet in recent studies with this drug this proportion has not exceeded 4%. Overall, there was an approximately 6-fold reduction in the proportion of patients with acute *P. falciparum* malaria who experienced recurrent *P. vivax* malaria during and after 2010, compared with the proportion before 2010.

These studies emphasize the importance of adequate follow-up to capture *P. vivax* recurrences when slowly eliminated antimalarial drugs are used. This is particularly important when comparing drugs with different elimination kinetics, such as artemether-lumefantrine and dihydroartemisinin-piperaquine. A 6-week follow-up period captures most of the recurrences following artemether-lumefantrine treatment but less than half of those that follow dihydroartemisinin-piperaquine therapy. Overall, half of *P. vivax* recurrences occurred between 6 and 9 weeks after *P. falciparum* malaria treatment. Indeed, 9 weeks of follow-up may be insufficient to capture all relapses, particularly after slowly eliminated treatments (ie, those containing piperaquine and mefloquine).

The remarkable recent substantial reduction in sequential infections in Myanmar presumably reflects a recent decline in *P. vivax* transmission. This is most likely explained by a reduction in the hypnozoite “bank” resulting from fewer infected inocula due to improved malaria control. It is not a result of a relative reduction in *P. vivax* transmission as compared to *P. falciparum* transmission, because reductions in *P. vivax* transmission usually lag slightly behind those of *P. falciparum* transmission. Indeed, the ratio of *P. vivax* to *P. falciparum* in both symptomatic and asymptomatic infections has increased. Radical cure with 8-aminoquinolines is an essential component of *P. vivax* malaria treatment, providing benefits to individuals and, in a low-level transmission setting, communities. Radical treatment with primaquine is part of national antimalarial treatment policy in Myanmar, but the proportion of all patients who receive this and take a full course is uncertain, so the contribution of primaquine to the reduction in population hypnozoite carriage and, thus, the dramatic decreases in recurrent *P. vivax* malaria following *P. falciparum* malaria is unclear.

Limitations of this analysis include the smaller sample size in the recent period (reflecting fewer cases) and the reliance on aggregate data. *P. vivax* malaria transmission is geographically heterogeneous, although some of the studies reported before and after the marked reduction in sequential *P. vivax* malaria were conducted in the same area. It is likely that, in areas where malaria control has not improved, there would still be high rates of *P. vivax* following *P. falciparum* malaria. In a previous analysis, the risk factors identified for *P. vivax* recurrence following *P. falciparum* malaria were mixed infection at enrollment, male sex, younger age, lower hematocrit, higher asexual *P. falciparum* parasite density, and *P. falciparum* gametocytemia on presentation [2]. Age may be a significant confounder in this overview, with more-recent studies enrolling a higher proportion of adults. ACTs with a longer-acting partner drug (eg, mefloquine and piperaquine) were also protective against relapse before 63 days. Nearly half the studies did not follow patients for >6 weeks. As described above, this leads to a substantial underestimation of what was previously the main complication of *P. falciparum* malaria in this region, namely recurrent *P. vivax* infection. Nevertheless, failure to control for these factors is unlikely to account for the substantial (>85%) decrease in relapse numbers observed here, for which the most plausible explanation is a reduction in *P. vivax* transmission intensity.

Overall, these findings illustrate the epidemiological usefulness of recording sequential *P. vivax* following *P. falciparum* malaria as a measure of transmission and emphasize the benefits of improved malaria control. Whereas there was a good case previously for a unified treatment policy providing a radical curative regimen for both *P. vivax* and *P. falciparum* malaria, this is no longer justified in areas where transmission has been reduced substantially.

## Supplementary Data

Supplementary materials are available at *The Journal of Infectious Diseases* online. Consisting of data provided by the authors to benefit the reader, the posted materials are not copyedited and are the sole responsibility of the authors, so questions or comments should be addressed to the corresponding author.

jiz052_suppl_Supplementary_Material

## Notes

***Acknowledgments.* ** We thank all the research teams involved in collecting the data; Isabelle Borghini from Medicines for Malaria Venture, for sharing individual patient data for one study; and the patients who participated to the studies.

***Financial support.* **This work was supported by the Mahidol-Oxford Tropical Medicine Research Unit, which is funded by the Wellcome Trust.

***Potential conflicts of interest.* **All authors: No reported conflicts of interest. All authors have submitted the ICMJE Form for Disclosure of Potential Conflicts of Interest. Conflicts that the editors consider relevant to the content of the manuscript have been disclosed.

## References

[1] Looareesuwan S, White NJ, Chittamas S, Bunnag D, Harinasuta T High rate of *Plasmodium vivax* relapse following treatment of falciparum malaria in Thailand. Lancet 1987; 2:1052–5. 2889965 10.1016/s0140-6736(87)91479-6

[2] Douglas NM, Nosten F, Ashley EA, et al. *Plasmodium vivax* recurrence following falciparum and mixed species malaria: risk factors and effect of antimalarial kinetics. Clin Infect Dis 2011; 52:612–20. 21292666 10.1093/cid/ciq249 PMC3060895

[3] Smithuis F, Kyaw MK, Phe O, et al. Effectiveness of five artemisinin combination regimens with or without primaquine in uncomplicated falciparum malaria: an open-label randomised trial. Lancet Infect Dis 2010; 10:673–81. 20832366 10.1016/S1473-3099(10)70187-0 PMC2947715

[4] Imwong M, Nakeesathit S, Day NP, White NJ A review of mixed malaria species infections in anopheline mosquitoes. Malar J 2011; 10:253. 21880138 10.1186/1475-2875-10-253 PMC3201030

[5] White NJ Determinants of relapse periodicity in *Plasmodium vivax* malaria. Malar J 2011; 10:297. 21989376 10.1186/1475-2875-10-297 PMC3228849

[6] Lysenko AJ, Beljaev AE, Rybalka VM Population studies of *Plasmodium vivax*. 1. The theory of polymorphism of sporozoites and epidemiological phenomena of tertian malaria. Bull World Health Organ 1977; 55:541–9. 338188 PMC2366697

[7] Commons RJ, Simpson JA, Thriemer K, et al. Risk of *Plasmodium vivax* parasitaemia after *Plasmodium falciparum* infection: a systematic review and meta-analysis. Lancet Infect Dis 2019; 19:91–101. 30587297 10.1016/S1473-3099(18)30596-6 PMC6300482

[8] Lacerda MVG, Bassat Q Primaquine for all: is it time to simplify malaria treatment in co-endemic areas? Lancet Infect Dis 2019; 19:10–2. 30587279 10.1016/S1473-3099(18)30612-1

[9] Imwong M, Boel ME, Pagornrat W, et al. The first *Plasmodium vivax* relapses of life are usually genetically homologous. J Infect Dis 2012; 205:680–3. 22194628 10.1093/infdis/jir806 PMC3266132

[10] Carrara VI, Lwin KM, Phyo AP, et al Malaria burden and artemisinin resistance in the mobile and migrant population on the Thai-Myanmar border, 1999–2011: an observational study. PLoS Med 2013; 10:e1001398. 23472056 10.1371/journal.pmed.1001398 PMC3589269

[11] WorldWide Antimalarial Resistance Network. WWARN Clinical Trials Publication Library http://www.wwarn.org/tools-resources/literature-reviews/wwarn-clinical-trials-publication-library. Accessed 18 February 2018.

[12] Smithuis FM, Monti F, Grundl M, et al. *Plasmodium falciparum*: sensitivity in vivo to chloroquine, pyrimethamine/sulfadoxine and mefloquine in western Myanmar. Trans R Soc Trop Med Hyg 1997; 91:468–72. 9373658 10.1016/s0035-9203(97)90288-1

