# Supplementary material for: Plasmodium vivax Relapse Rates Following Plasmodium falciparum Malaria Reflect Previous Transmission Intensity
Source: J Infect Dis. 2019 Jan 29;220(1):100–4. doi: 10.1093/infdis/jiz052 (PMC6548896; doi:10.1093/infdis/jiz052)
Supplement: jiz052_suppl_Supplementary_Material [file jiz052_suppl_supplementary_material.docx]

**Supplementary References**

1. Ashley EA, McGready R, Hutagalung R, et al. A randomized, controlled study of a simple, once-daily regimen of dihydroartemisinin-piperaquine for the treatment of uncomplicated, multidrug-resistant falciparum malaria. Clin Infect Dis 2005; 41:425-32.
2. Smithuis F, Kyaw MK, Phe O, Aye KZ, Htet L, Barends M, Lindegårdh N, Singtoroj T, Ashley E, Lwin S, Stepniewska K, White NJ. Efficacy and effectiveness of dihydroartemisinin-piperaquine versus artesunate-mefloquine in falciparum malaria: an open-label randomised comparison. Lancet 2006; 367:2075-85.
3. Ashley EA, Lwin KM, McGready R, Simon WH, Phaiphun L, Proux S, Wangseang N, Taylor W, Stepniewska K, Nawamaneerat W, Thwai KL, Barends M, Leowattana W, Olliaro P, Singhasivanon P, White NJ, Nosten F. An open label randomized comparison of mefloquine-artesunate as separate tablets vs. a new co-formulated combination for the treatment of uncomplicated multidrug-resistant falciparum malaria in Thailand. Trop Med Int Hlth 2006; 11: 1653-60.
4. Valecha N, Phyo AP, Mayxay M, Uthaisin C, Newton PN, Krudsood S, Keomany S, Khanthavong M, Pongvongsa T, Ruangveerayuth R, Uthaisil C, Ubben D, Duparc S, Bacchieri A, Corsi M, Rao BH, Bhattacharya PC, Dubhashi N, Ghosh SK, Dev V, Kumar A, Pukrittayakamee S. An open-label, randomised study of dihydroartemisinin-piperaquine versus artesunate-mefloquine for falciparum malaria in Asia. PloS One 2010; 5: e11880.
5. Rueangweerayut R, Phyo AP, Poravuth Y, Binh TQ, Tinto H, Pénali LK, Valecha N, Tien NT, Abdulla S, Borghini-Fuhrer I, Duparc S, Shin CS, Fleckenstein L; Pyronaridine–Artesunate Study Team. Pyronaridine-artesunate versus mefloquine plus artesunate for malaria. N Engl J Med 2012; 366:1298-309.
6. Na-Bangchang K, Ruengweerayut R, Mahamad P, Ruengweerayut K, Chaijaroenkul W. Declining in efficacy of a three-day combination regimen of mefloquine-artesunate in a multi-drug resistance area along the Thai-Myanmar border. Malaria J 2010; 9: e273.
7. Phyo AP, Ashley EA, Anderson TJC, Bozdech Z, Carrara VI, Sriprawat K, Nair S, White MM, Dziekan J, Ling C, Proux S, Konghahong K, Jeeyapant A, Woodrow CJ, Imwong M, McGready R, Lwin KM, Day NPJ, White NJ, Nosten F. Declining Efficacy of Artemisinin Combination Therapy Against *P. Falciparum* Malaria on the Thai-Myanmar Border (2003-2013): The Role of Parasite Genetic Factors. Clin Infect Dis 2016; 63: 784-91.
8. Kyaw MP, Nyunt MH, Chit K,  Aye MM, Aye KH, Aye MM, Lindegardh N, Tarning J, Imwong M, Jacob CG, Rasmussen C, Perin J, Ringwald P, Nyunt MM. Reduced susceptibility of Plasmodium falciparum to artesunate in southern Myanmar. PloS One 2013; 8:e57689.
9. Ashley EA, Dhorda M, Fairhurst RM, Amaratunga C, Lim P, Suon S, Sreng S, Anderson JM, Mao S, Sam B, Sopha C, Chuor CM, Nguon C, Sovannaroth S, Pukrittayakamee S, Jittamala P, Chotivanich K, Chutasmit K, Suchatsoonthorn C, Runcharoen R, Hien TT, Thuy-Nhien NT, Thanh NV, Phu NH, Htut Y, Han KT, Aye KH, Mokuolu OA, Olaosebikan RR, Folaranmi OO, Mayxay M, Khanthavong M, Hongvanthong B, Newton PN, Onyamboko MA, Fanello CI, Tshefu AK, Mishra N, Valecha N, Phyo AP, Nosten F, Yi P, Tripura R, Borrmann S, Bashraheil M, Peshu J, Faiz MA, Ghose A, Hossain MA, Samad R, Rahman MR, Hasan MM, Islam A, Miotto O, Amato R, MacInnis B, Stalker J, Kwiatkowski DP, Bozdech Z, Jeeyapant A, Cheah PY, Sakulthaew T, Chalk J, Intharabut B, Silamut K, Lee SJ, Vihokhern B, Kunasol C, Imwong M, Tarning J, Taylor WJ, Yeung S, Woodrow CJ, Flegg JA, Das D, Smith J, Venkatesan M, Plowe CV, Stepniewska K, Guerin PJ, Dondorp AM, Day NP, White NJ; Tracking Resistance to Artemisinin Collaboration (TRAC). Spread of artemisinin resistance in Plasmodium falciparum malaria. N Engl J Med 2014; 371: 411-23.
10. Wang Y, Yang Z, Yuan L, Zhou G, Parker D, Lee MC, Yan G, Fan Q, Xiao Y, Cao Y, Cui L. Clinical Efficacy of Dihydroartemisinin-Piperaquine for the Treatment of Uncomplicated Plasmodium falciparum Malaria at the China-Myanmar Border. Am J Trop Med Hyg 2015; 93: 577-83.
11. Tun KM, Jeeyapant A, Imwong M, Thein M, Aung SS, Hlaing TM, Yuentrakul P, Promnarate C, Dhorda M, Woodrow CJ, Dondorp AM, Ashley EA, Smithuis FM, White NJ, Day NP. Parasite clearance rates in Upper Myanmar indicate a distinctive artemisinin resistance phenotype: a therapeutic efficacy study. Malaria journal 2016; 15:185.
12. Tun KM, Jeeyapant A, Myint AH, Kyaw ZT, Dhorda M, Mukaka M, Cheah PY, Imwong M, Hlaing T, Kyaw TH, Ashley EA, Dondorp A, White NJ, Day NPJ, Smithuis F. Effectiveness and safety of 3 and 5 day courses of artemether-lumefantrine for the treatment of uncomplicated falciparum malaria in an area of emerging artemisinin resistance in Myanmar. Malaria J 2018; 17: e258.
